# Supplementary material for: Spatial specificity of auxin responses coordinates wood formation
Source: Nat Commun. 2018 Feb 28;9:875. doi: 10.1038/s41467-018-03256-2 (PMC5830446; doi:10.1038/s41467-018-03256-2)
Supplement: Supplementary file 1 — Supplementary Information [file 41467_2018_3256_MOESM1_ESM.pdf]

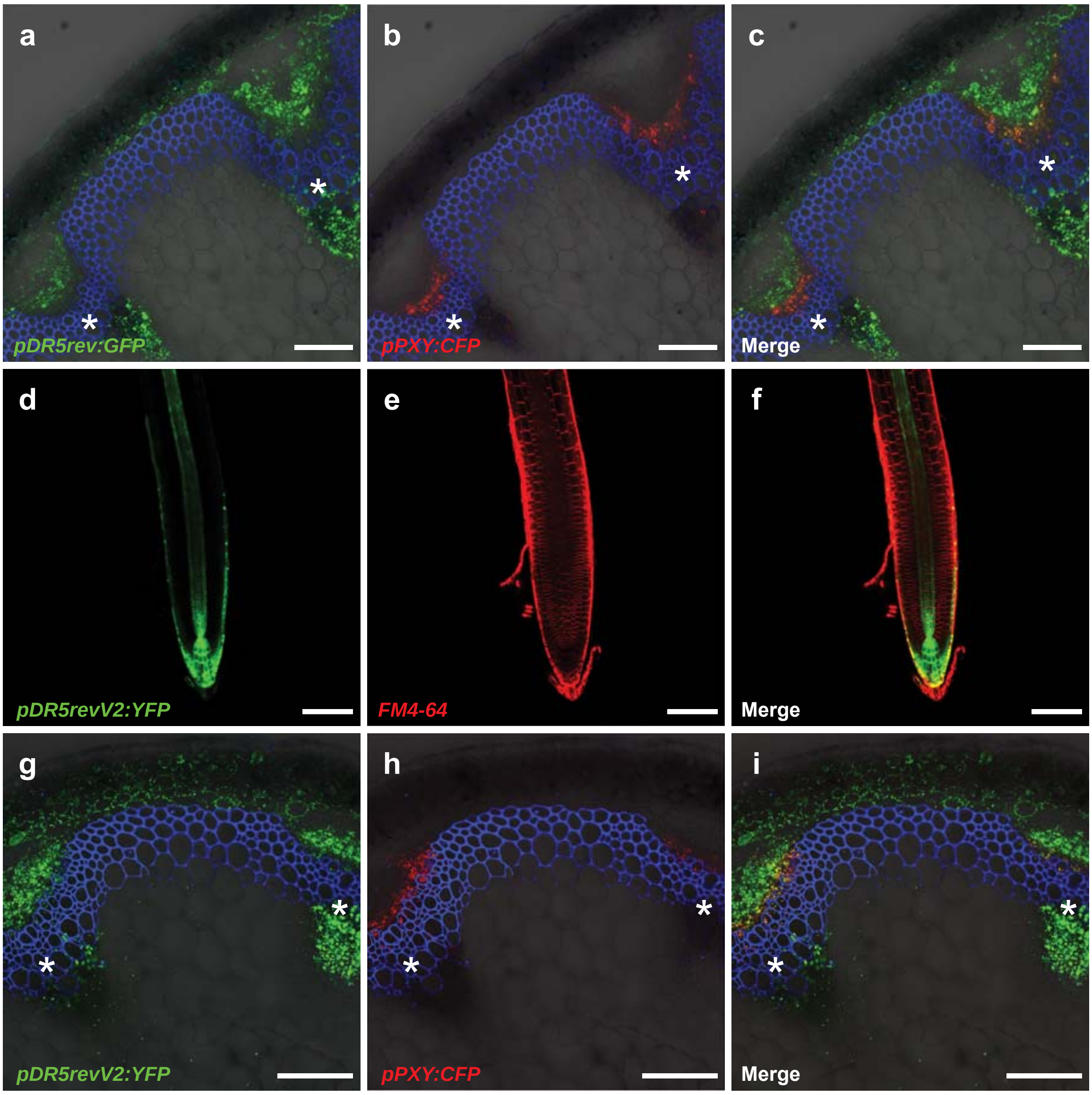

Supplementary Figure 1:

a-c: Confocal analyses of cross sections from the second internode (primary stem) of plants containing the auxin response markers *pDR5rev:GFP* and the stem cell marker *pPXY:CFP*. Asterisks mark the vascular bundles. Size bars represent 100 µm. PI staining in blue.
d-f: Confocal analyses of root tips of 7-day-old seedlings carrying the *pDR5revV2:YFP* reporter. Size bars represent 100 µm. FM4-64 staining in red.
g-i: Confocal analyses of cross sections from the second internode (primary stem) of plants containing the auxin response markers *pDR5revV2:YFP* and the stem cell marker *pPXY:CFP*. Asterisks mark the vascular bundles. Size bars represent 100 µm. PI staining in blue.

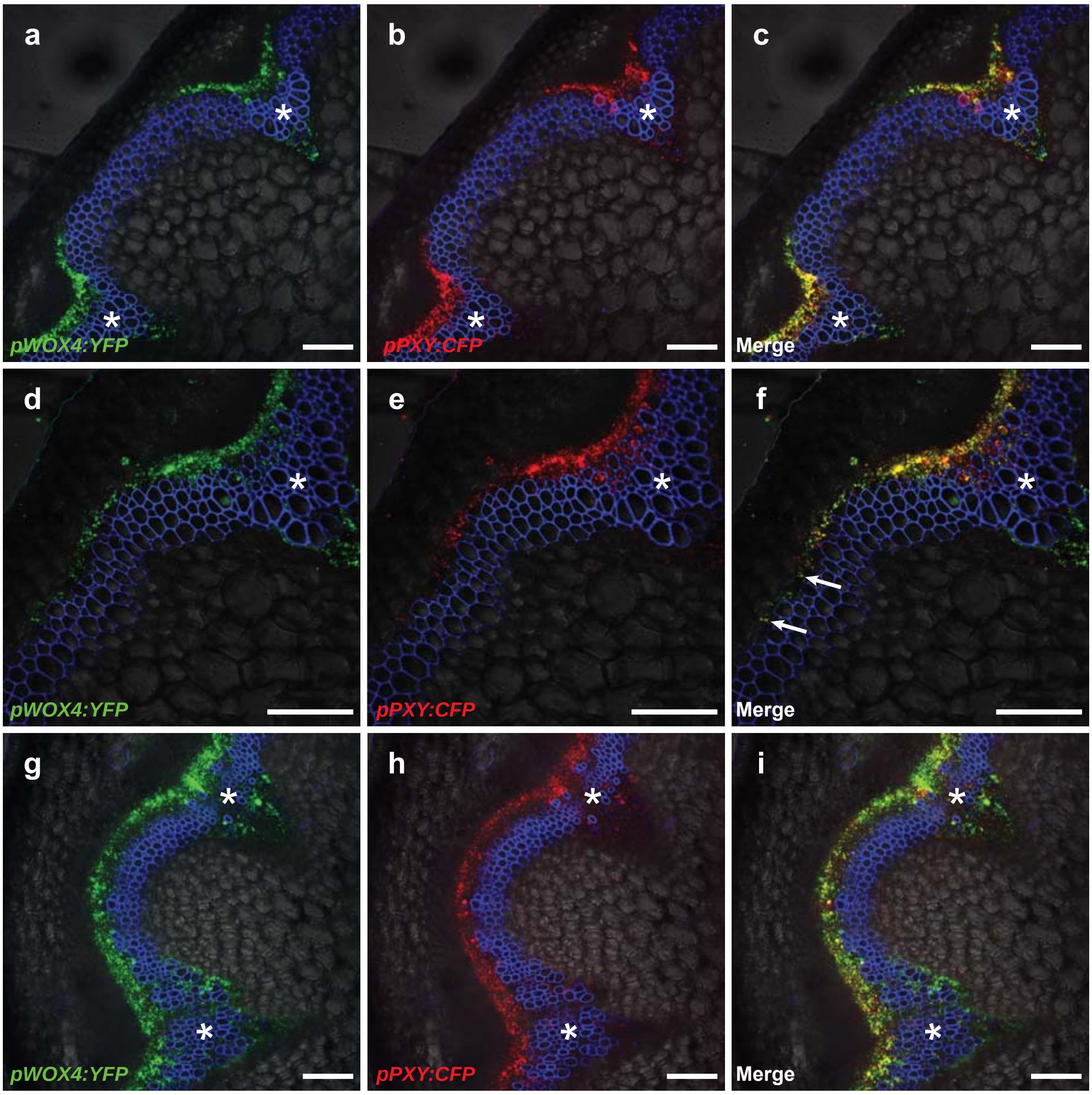

Supplementary Figure 2:

a-i: Confocal analyses of cross sections from the second internode (a-c), 5 mm above the stem base (d-f) and the stem base (g-i) of plants containing *pWOX4:YFP* and *pPXY:CFP*. Asterisks mark vascular bundles. Size bars represent 100 µm. PI staining in blue.

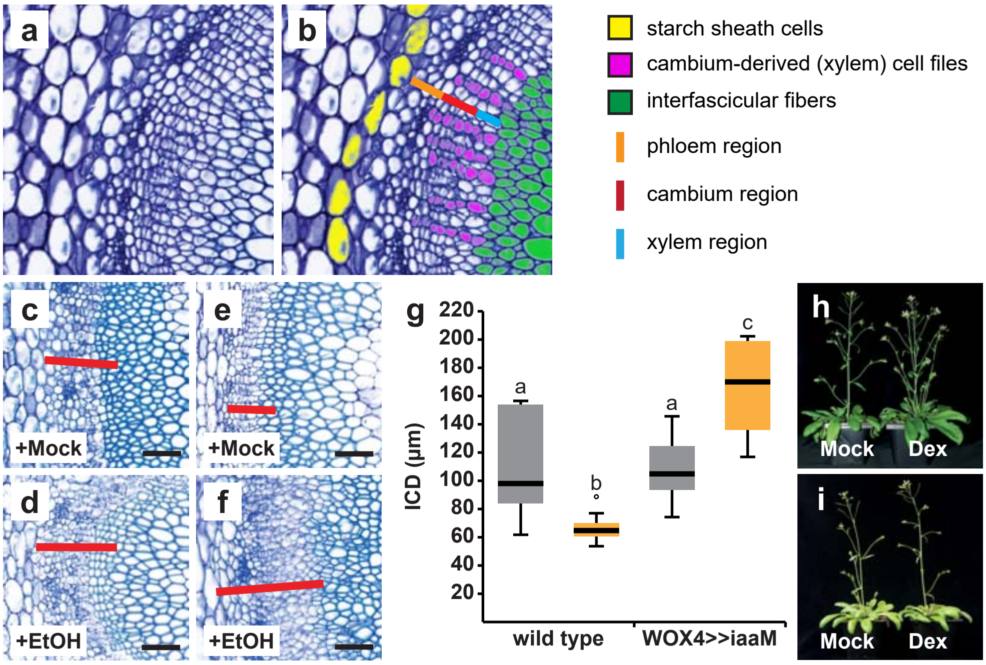

Supplementary Figure 3:

a,b: Indication of the quantification strategy of the cambium width and the width of ICD tissues. The same section is shown without (a) and with (b) colour codings. The starch sheath (characteristic cells labelled in yellow), which is the innermost cell layer of the cortex, was used to define the outer boundary of the ICD tissues. The proximal end of cell files possible to identify along the cambium area were used to define the inner boundary of ICD tissues. Note that a combination of several cell files was taken into consideration in this case. Interfascicular fibers, which do not originate from the cambium, were identifyed based on their thicker cell wall and the fact that the are not organized in radial cell files. The cambium region was definded as the region with small and undiferentiated cells, the xylem as the tissue between cambium and interfascicular fiber cells and the phloem as the region beween cambium and starch sheath cells.
c-f: Toluidine blue stained cross sections from the stem base of wild type (c, d) and *pWOX4:AlcR; pAlcA:iaaM* (e, f), plants after long-term EtOH (d, f) or mock (c, e) treatment. Interfascicular regions are shown and the whole of cambium and ICD tissues are marked (red bar). Size bars represent 50 µm.
g: Quantification of ICD tissue extension at the stem base of wild type and *pWOX4:AlcR; palcA:iaaM* (*WOX-4>>iaaM*) plants after long-term EtOH (yellow), mock (light grey) treatment. Statistical groups indicated by letters were determined by one-way ANOVA with post hoc Tamhane-T2 (CI 95%, Sample size n=11-14).
h,i: Overview pictures of 15-20 cm tall *pPXY:Myc-GR-bdl* (h) and *pBDL:Myc-GR-bdl* (i) plants after long-term mock or Dex treatments, respectively.

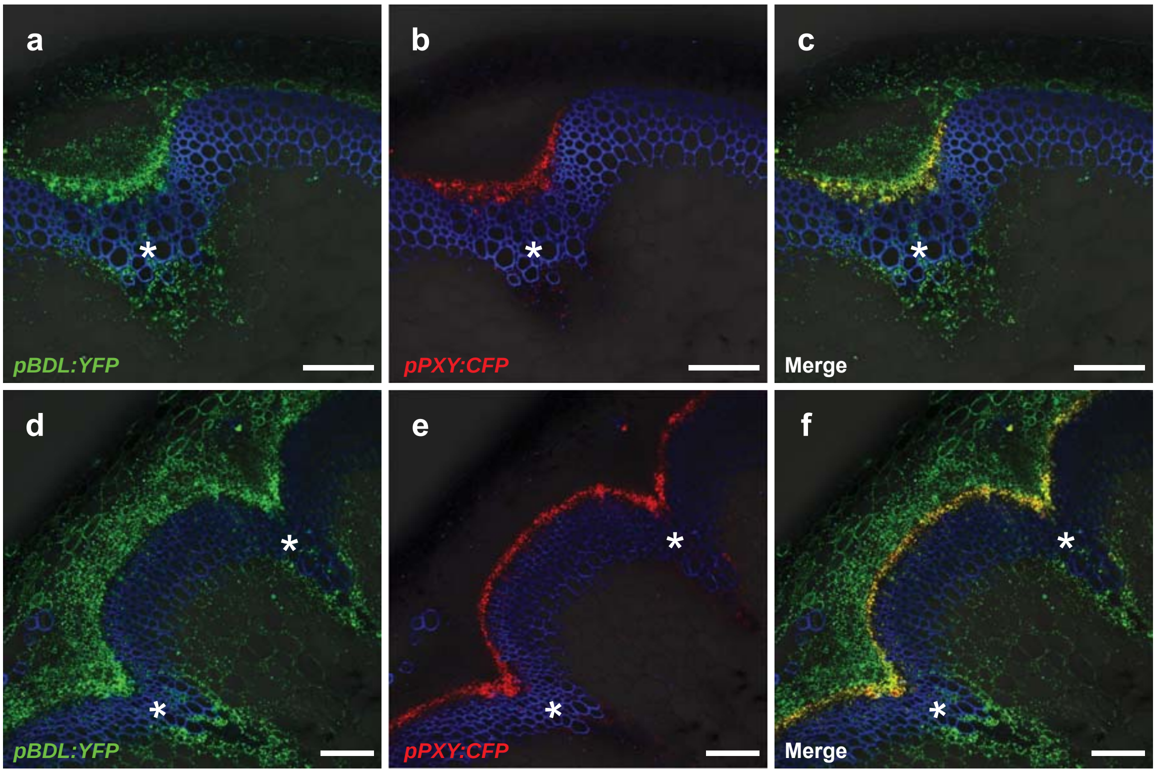

Supplementary Figure 4:

a-f: Confocal analyses of cross sections from the second internode (a-c) and stem base (d-f) of plants carrying *pBDL:YFP* and the stem cell marker *pPXY:CFP*. Asterisks mark vascular bundles. Size bars represent 100 µm. PI staining in blue.

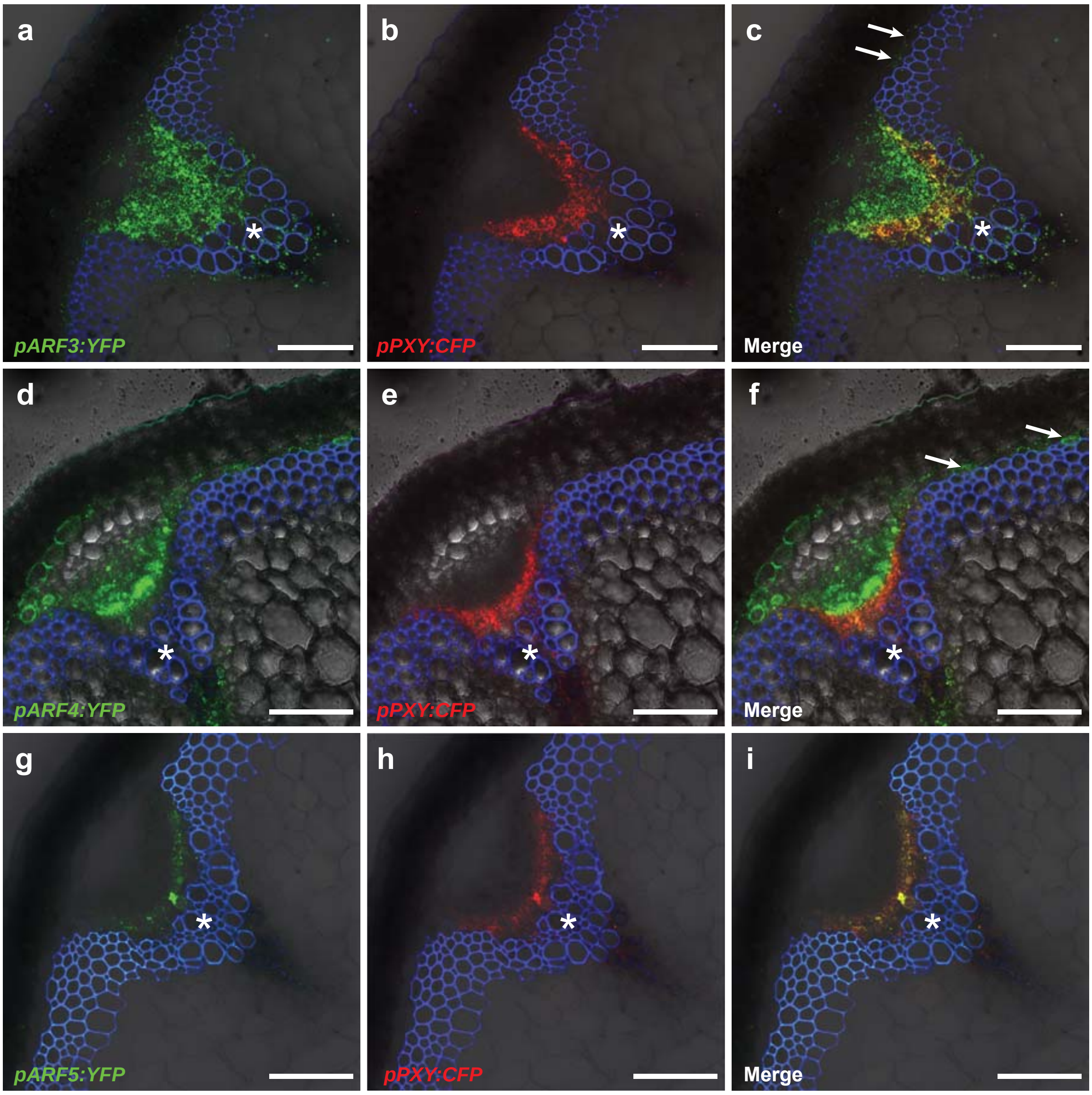

Supplementary Figure 5:

a-i: Confocal analyses of second internodes of plants containing *pARF3:YFP* (a-c), *pARF4:YFP* (d-f) and *pARF5:YFP* (g-i), respectively, and the stem cell marker *pPXY:CFP*. Arrows mark the expression of *pARF3:YFP* (c) and *pARF4:YFP* (f) in interfascicular region (starch sheath). Asterisks mark the vascular bundles. Scale bars represent 100 μm. PI staining in blue.

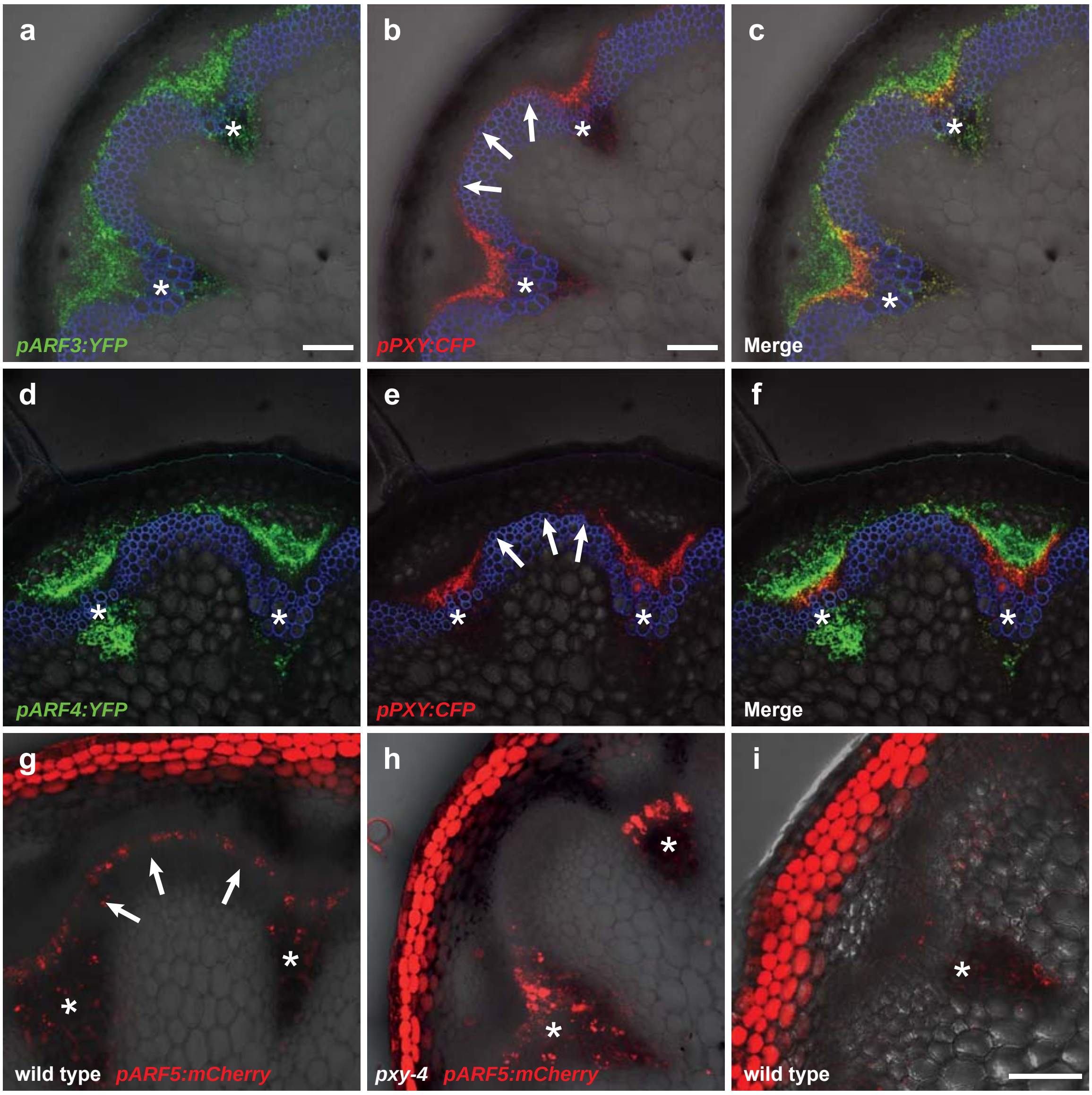

Supplementary Figure 6:

a-f: Confocal analyses of cross sections 5 mm above the stem base (transition zone) of plants containing *pARF3:YFP* (a-c) and *pARF4:YFP* (d-f), respectively, and the stem cell marker *pPXY:CFP*. Arrows indicate *pPXY:CFP* activity in interfascicular regions. Asterisks mark the vascular bundles. Size bars represent 100 µm. PI staining in blue.
g-i: Confocal analyses of cross sections at the stem base from wild type and *pxy-4* mutant plants containing the *pARF5:mCherry* reporter. Asterisks mark the vascular bundles. Arrows indicate *pARF5:mCherry* activity in interfascicular regions. i shows auto-fluorescence at the stem base of wild type plants imaged with the same settings as in g and h. Size bars represent 100 µm.

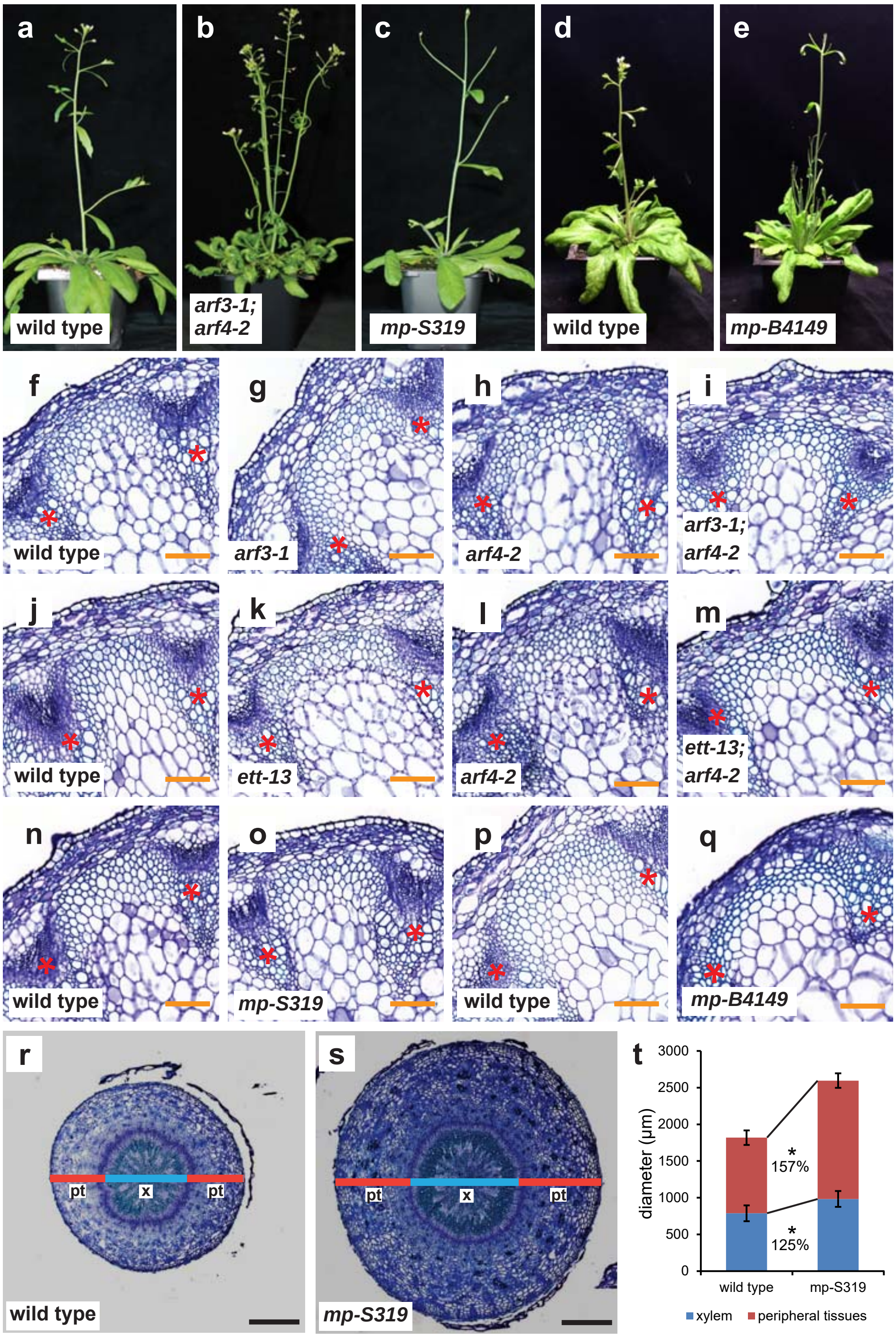

Supplementary Figure 7:

a-c: Growth habitus of wild type, *arf3-1;arf4-2* and *mp-S319* mutants.
d,e: Growth habitus of wild type and *mp-B4149* after tissue culture-based rooting.
f-m: Primary stem conformation 9 mm above the stem base in *arf3* and *arf4* single and double mutants in comparison to wild type.
n-q: Primary stem conformation 9 mm above the stem base in *arf5* mutants in comparison to wild type.
r-t: Radial extension of wild type (r, t) and *mp-S319* hypocotyls (s, t). Student's T-test was performed comparing wild type and *mp-S319* (xylem: p=0.0001; peripheral tissues: p=2.1E-11) (Sample sizes: n=10-13). Significance is indicated by asterisks. Size bars represent 100 µm (f-q) and 500 µm (r,sS). Asterisks in f-q indicate primary vascular bundles. pt = peripheral tissues, x = xylem

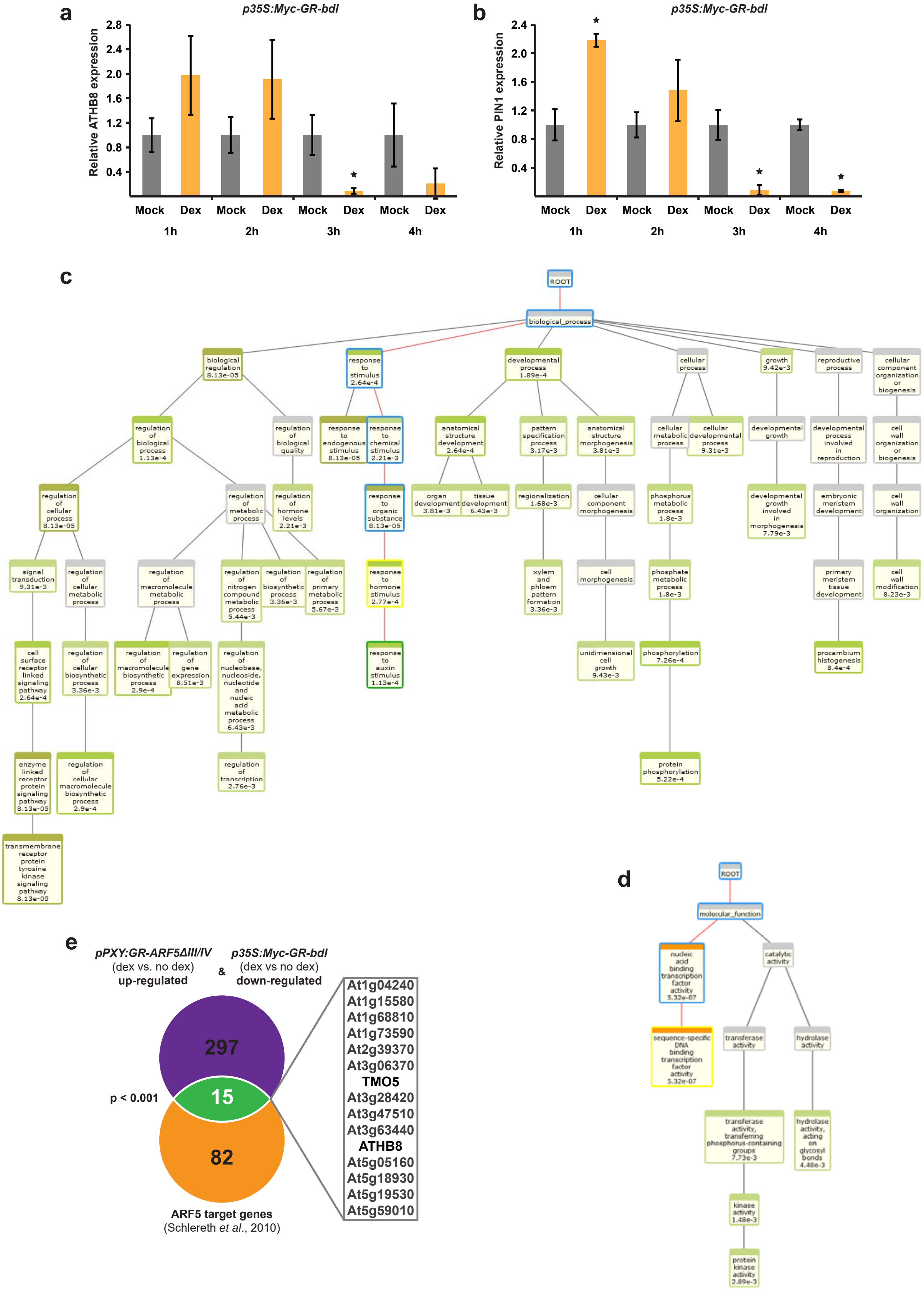
a
p35S:Myc-GR-bdl
Relative ATHB8 expression
2.8
2.4
2.0
1.6
1.2
0.8
0.4
Mock Dex Mock Dex Mock Dex Mock Dex
1h 2h 3h 4h
b
p35S:Myc-GR-bdl
Relative PIN1 expression
2.4
2.0
1.6
1.2
0.8
0.4
Mock Dex Mock Dex Mock Dex Mock Dex
1h 2h 3h 4h
c
ROOT
biological_process
biological regulation 8.13e-05
response to stimulus 2.64e-4
developmental process 1.89e-4
cellular process
growth 9.42e-3
reproductive process
cellular component organization or biogenesis
regulation of biological process 1.13e-4
regulation of biological quality
response to endogenous stimulus 8.13e-05
response to chemical stimulus 2.21e-3
anatomical structure development 2.64e-4
pattern specification process 3.17e-3
anatomical structure morphogenesis 3.81e-3
cellular metabolic process
cellular developmental process 9.31e-3
developmental growth
developmental process involved in reproduction
cell wall organization or biogenesis
regulation of cellular process 8.13e-05
regulation of metabolic process
regulation of hormone levels 2.21e-3
response to organic substance 8.13e-05
organ development 3.81e-3
tissue development 6.43e-3
regionalization 1.68e-3
cellular component morphogenesis
phosphorus metabolic process 1.8e-3
developmental growth involved in morphogenesis 7.79e-3
embryonic meristem development
cell wall organization
signal transduction 9.31e-3
regulation of cellular metabolic process
regulation of macromolecule metabolic process
regulation of nitrogen compound metabolic process 5.44e-3
regulation of biosynthetic process 3.36e-3
regulation of primary metabolic process 5.67e-3
response to hormone stimulus 2.77e-4
xylem and phloem pattern formation 3.36e-3
cell morphogenesis
phosphate metabolic process 1.8e-3
primary meristem tissue development
cell wall modification 8.23e-3
cell surface receptor linked signaling pathway 2.64e-4
regulation of cellular biosynthetic process 3.36e-3
regulation of macromolecule biosynthetic process 2.9e-4
regulation of gene expression 8.51e-3
regulation of nucleobase, nucleoside, nucleotide and nucleic acid metabolic process 6.43e-3
response to auxin stimulus 1.13e-4
unidimensional cell growth 9.43e-3
phosphorylation 7.26e-4
procambium histogenesis 8.4e-4
enzyme linked receptor protein signaling pathway 8.13e-05
regulation of cellular macromolecule biosynthetic process 2.9e-4
regulation of transcription 2.76e-3
protein phosphorylation 5.22e-4
transmembrane receptor protein tyrosine kinase signaling pathway 8.13e-05
d
ROOT
molecular_function
nucleic acid binding transcription factor activity 5.32e-07
catalytic activity
sequence-specific DNA binding transcription factor activity 5.32e-07
transferase activity
hydrolase activity
transferase activity, transferring phosphorus-containing groups 7.73e-3
hydrolase activity, acting on glycosyl bonds 4.48e-3
kinase activity 1.48e-3
protein kinase activity 2.89e-3
e
pPXY:GR-ARF5ΔIII/IV
(dex vs. no dex)
up-regulated
&
p35S:Myc-GR-bdl
(dex vs no dex)
down-regulated
297
15
82
p < 0.001
ARF5 target genes
(Schlereth et al., 2010)
At1g04240
At1g15580
At1g68810
At1g73590
At2g39370
At3g06370
TMO5
At3g28420
At3g47510
At3g63440
ATHB8
At5g05160
At5g18930
At5g19530
At5g59010

Supplementary Figure 8:

a,b: Optimization of the duration of Dex treatment in the second internode of *p35S:Myc-GR-bdl* plants. Shown are the analyses of transcript levels of *ATHB8* (a) or *PIN1* (b) by quantitative RT-PCR. Error bars: ± standard deviation. Student's T-test (*ATHB8* 1h p=0.07, 2h p=0.11, 3h p=8.69E-03, 4 h p=0.19 and *PIN1* 1h p=9.86E-04, 2h p=0.06, 3h p0= 4.98E-03, 4H p=5.03E-04) was performed comparing mock and Dex treated samples (Sample sizes n=2-3). Significance is indicated by the asterisk.

c,d: BioMaps analysis of biological process (c) and molecular function (d) of genes modulated by *p35S:Myc-GR-bdl* and *pPXY-GR-ARF5ΔIII/IV* induction (600 genes, p-value < 0.05, Supplementary Data 2) by Virtual Plant 1.3 with a cut-off p-value < 0.01 and the *Arabidopsis thaliana* Columbia (TAIR10) genome as background population.

e: Comparison of genes induced by *pPXY-GR-ARF5ΔIII/IV* and repressed by *p35S:Myc-GR-bdl* induction and a dataset of putative ARF5 target genes. Non-random degree of overlap was tested by using VirtualPlant 1.3 GeneSect with a cut-off p-value < 0.05 and the *Arabidopsis thaliana* Columbia (TAIR10) genome as background population.

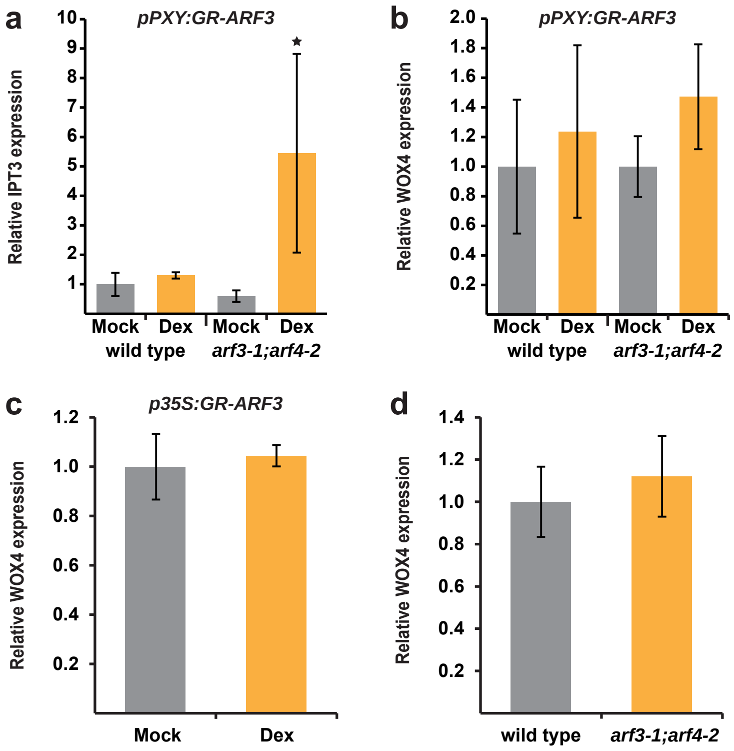

Supplementary Fig. 9:

a,b: Analysis of *IPT3* (a) and *WOX4* (b) transcript levels by quantitative RT-PCR in the second internode of wild type and *arf3-1;arf4-2* mutant plants expressing *pPXY:GR-ARF3* after 3 hour mock (grey) or Dex (yellow) treatment.
c: Analysis of *WOX4* transcript levels by quantitative RT-PCR in the second internode of wild type plants expressing *p35S:GR-ARF3* after 3 hour mock (grey) or Dex (yellow) treatment.
d: Analysis of *WOX4* transcript levels by quantitative RT-PCR at the stem base of wild type and *arf3-1;arf4-2* mutant plants.
Error bars: ± standard deviation. Student's T-test (*pPXY:GR-ARF3 IPT3* p=0.17 & *WOX4* p=0.47, *pPXY:GR-ARF3;arf3-1;arf4-2 WOX4* p=0.17, *p35S:GR-ARF3 WOX4* p=0.61, wild type and *arf3-1;arf4-2 WOX4* p=0.45) or Welch's T-test (*pPXY:GR-ARF3;arf3-1;arf4-2 IPT3* p=4.85E-02) were performed comparing mock and Dex and wild type and mutant, respectively (Sample size n=3-4). Significance is indicated by asterisk.

a

```
>chr1 17237952 17242053 - AT1G46480.1

                                                            tttggttcatttggttttgggagcggattaaaacgtagaaactacg -2800
gtttcacaaacaacttatttaattttaagtaaagagaaaataactcctttttcttgaaatcttgataaaaaaaacaatcaattgcaatggtttatgttcct -2700
tgaataagaagttagagtttgttcaaaaacaaacaaaacctaaggcaaacctttgttcggttctcgatcagtcgatttatatgaatggatttctattcaa -2600
catatgttctcttttcttatttttcggccttgaatctcttatttttctatttttttcgggtcctcaagattatccacctagatgtgtctcactcttttcat -2500
ttccctattgcaggtcaacgacgttttatttaagctttttcccatagaccgattacccatttatcagaattacttggattaatggaattattctcgattt -2400
aagtcgaatttttcgaagggtgctacatatctcatcaacaaacaatacaaatcaaaggaaggaagaagagctaaagagctaaaaaaaagatgaggaggag -2300
atcgaagtaaacaaattggaagaaaaagagatccaacaatgattggttgtgaacatgttatgctagtttatttcaaattttgaaaccctaaacctcaaac -2200
catcagtaatcTGTCATtaCCGACAtagtattacatatggatcgaaaaataaacatgttaaatatgtgtcctaaaaaaatacatatgaacagtggtagag -2100
gcaagtgtagtggaggagggtcactgcatatgaatatcgacgatatagtatgggccatagataattgaagaaataaccatctgatcacagcttgacacac -2000
gaatcaggttatccatcgtgtgatgacatttttcaaaaaatatgaatggtattaccagtttcttgttatgctttgggttcagtaaagttccactcatttt -1900
ccagtatctgatgaatgatagagcagtctttttagaactttgagctttcatattccttccgtccaacaaagatgaattttctatggaaaaaaattgtccc -1800
acaaagatacaaattttgtattttaatataaattttaaaattgtttacctgttttatcctaagtaaataagtagataaatatttttttattattttaaa -1700
atatattaaaaggttaaaattgtcagtatacattatttttttaaaaatgtgaaatttctataaaaaaatccatcttcataggataaagatgttattaattt -1600
tagctattagtcatgatcaacctacactggtacttctatatttcctccatgatatcaatttttatgttttaaccagtgatatgaactgtgactttccacc -1500
ttcataactttgatatccatatatattagtgtaagtttcgtactgtacatatacttgcatcataactggattagccgagactaaataattttgactattc -1400
ttcacagttcatactcaaggtctctgttctttgtaattctagactacatatcaaataaaaagatgatgatatattagtcaaaactcaaaagtaattagaa -1300
gaaaaaactttgtaattccggaatatcatacgttactacttttcattagtaaaatacccaaattacttgggaaattgaggttctgcagttcccgatgtac -1200
aagagaataacaatacaatacactgatctaaagtgaatgggacgcttgacattgccaaaccaaaccatgcttttgtatcaatatctaataaacatataag -1100
cttaaatttataatgatgtattattatcaaaataaacaatgataatataacattaagatatttctagccttttcttttccgcataaaagtctgtcctggc -1000
tcattataaactaccactaatttaggcaaaatccgtatcttaacaaaagtaataaaagcaatttcgagtattgggattggaagatacctttttccccttat -900
tcattcttcttctcattcttcttttctctaaataaagtagttgagacttgagagccaccgaaaagcattcgacatcctatcattaaagctaactttttttc -800
agtgttttatttctaaaataatttcttaaaataaagaaaggtgagagagaaattatgtccctatacaactagtagaatgaaagattgtcaagctttggtt -700
tgggattttttctaataaatcacatgcatttgttagaatttgatcccaaaaaaaaaaaaattcaaaccttttttcctgtaaaagcaaacctattggctcgtag -600
gtcaatgtcaatctcaatgaatagtaaagtcttttgatatcacgtatctataacatattatttttagcgtggttcatgtccctcaacattctgcctaaat -500
tataggatctcaagacatagtactatagactacaactacaagtagaggaaaattttataagagagataaattatgcggtattcacgcatgacatgcgtaa -400
gcatgaaactaaaatttggttgatacattactatgccgaatttaaagtagtgctccatgCAGACAtgaacatatcagatatcacacgtacgcatgtatta -300
cgtgtatgtagagacacacgtacgcatatctatattcacacaatgagatttcactttctcaaacaaagatcactgtcttcaattagtttcaaatttgttc -200
tttacctattgcttttacatttctgagtgtatatatttacaataaggacaaggagattttaaatgataaaagtcaaccatatcccatcatttggtctttg -100
gtttcccgacgtcccacttttgtaaaaatcatctgttcattttccattctttttctttttcctacccaactggatatcaccaatgatatcattatacact +1
aacactatcatttcacacttatcttcctctatatatacatagcttaagtcttgtaacgagaaaggcatgcatagcatttgctagttttaacatatagcaATG
```

b

Matrix for ARF binding sites prediction	Position relative TSS	
	-2175	-335
M0147_1.02 (see ref.[77])	p-value < 0.0002	p-value < 0.0008
ARF_ARF2_col_v31 (see ref.[78])	p-value < 7e-05	p-value < 0.0002
ARF_ecoli_MP_col (see ref.[78])	p-value < 0.0002	p-value < 0.0002

Supplementary Fig. 10:

a: 3000 bp upstream of the *WOX4* start codon used for ARF binding site prediction. Identified sites are highlighted in yellow. The transcriptional start site (TSS) is indicated by +1.
b: The significances (p-values) of top-scored potential ARF binding sites located upstream of the start codon of the *WOX4* gene. The first column lists the frequency matrices that were used for ARF binding site prediction (see Methods). The other columns show the significances of potential hits estimated by p-values in relation to the score of the matrix.

Supplementary Table 1. Oligonucleotides used in this study

Oligo Name	Sequence (5'-3')
**Genotyping**	
SAIL_1211_F06_LP	ATGAAGTTTGACCAGCTTGCC
SAIL_1211_F06_RP	GGGGATTTTGATTTCGTAGGC
SALK_040513_LP	AACGGTAGTCCAAATCTTCGC
SALK_040513_RP	TCAGAAAAACAGAACAAAACCC
SALK_070506_LP	GGAGTCTGTGACTTGGACCC
SALK_070506_RP	TCATCATACCCGTTAAGTCGC
SALK_021319_LP	TCTTCCTTCCAGTCTCTTGCC
SALK_021319_RP	TTAAGATCGTTAATGCCTGCG
MP_for8	TCGGTGTTCTTGCTGCTG
MP_rev8	GGATGGAGCTGACGTTTG
Gabi_462G01_LP	TTTTTAGCGTGGTTCATGTCC
Gabi_462G01_RP	CATTTTTCCCTTCGATTTTCC
PXYfor7	TTTCCCTCGACCTCTCTCAC
PXYrev7	CCGTTCTCTTTGTTTTTTCCCC
**Promoters**	
WOX411for	ACTAGCGGCCGCTTTGGTTCATTTGGTTTTGGGA
WOX4rev9	ACTAGCTAGCTATATGTTAAAACTAGCAAATGC
At4g24550_for1	ACTAGGATCCTGTTTTCAGATAATGTTTATCCTTC
At4g24550_rev1	ACTACTCGAGATCGTTGCACCTTTATTTC
BDL_for2	ACTAGCGGCCGCCATGTGGTAGTGTCG
BDL_rev4	TCTAGATACCATGGTCAATAACAAAACCCTAGACTCC
BDL_for3	ACTACCCGGGCTTCCCTTCCAAAGC
BDL_rev5	ACTAGGTACCTTTCTAGGTAACAAGTTAAATATTACCTAAACAATCC
ARF3for1	ACTAGCGGCCGCGTCATTACCATTAAAGTCATC
ARF3rev1	ACTAGGATCCATCCATGGTAAAGAGAGAGAAACAGAGATAAAG
ARF3for2	ACTAGGATCCATCTGCAGAAGGGTTCTTTGGTTTCTGTG
ARF3rev2	ACTAGGTACCCATTGGCTTGGATTACGTTTTC
ARF4for1	ACTAGCGGCCGCACACCAACTCTGTTCAACTCT
ARF4rev1	ATCAACTAGTTGAAAAAGCTTCTCTTTTAAGAG
ARF4for2	ACTACCCGGGTACCCATAAAGAAGCTTATTTC
ARF4rev2	ACTAGGTACCACAGATATTGTGAAATATTCC
MP_for7	ACTAGCGGCCGCAGATCATCTTGAACGGTAATCG
MP_rev5	ACTAGGATCCTACATATGACAGAGAGATTTTTCAATGTTC
MP_for5	ACTAGGATCCTACTCGAGATGTAACAATATAAAAATGATC
MP_rev6	ACTAGGGCCCTGCAGAATTAGCATACCACACATGC
AT4G32880_for_2	GGGAACAAAAGCTGGAAACGGTAGGAAATAGATTCACAGA
AT4G32880_rev_2	GGCCCGGGATCCATGCTTTGATCCTCTCCGATCTCTC
RULfor2	ACTACTCGAGGGATTTCATTAAATCTAAATGG
RULrev2	ACTAGAATTCATCATATGGGTTGAGCTATTTGGTGACTCAC
RULfor3	ACTAACTAGTTTCCTCATTCCGACCAAAAG
RULrev3	ACTAGCGGCCGCATATTTTTTTGTTTCTGTAG

ORFs	
Myc_for1	ACTATCTAGAATGGGGGAGCAAAAGCTTATTTCTG
BDL_rev3	ACTAGGATCCTAAATAGGGTTGTTTCTTTGTCTATCC
Myc_for5	ACTATCATGATAGAGCAAAAGCTTATTTCTGAGGAGG
BDL_rev7	ACTACCCGGGGATCCTAAATAGGGTTG
GR_for1	ACTATCTAGAATGATTCAGCAAGCCACTGCAGG
GR_rev3	ACTACCCGGGTAGTCGACTTTTTGATGAAACAGAAGC
ARF3_for4	ACTAGTCGACATGGGTGGTTTAATCGATCTG
ARF3_rev4	ACTACCCGGGCTAGAGAGCAATGTCTAGC
MP_for16	ACTAGTCGACATGATGGCTTCATTGTCTTGTG
MP_rev14	ACTAGAATTCTTATGAAACAGAAGTCTTAAGATCGTTAATGCC
GR_for5	ACTATCATGATTCAGCAAGCCACTGCAGGAGTCTC
MP_for18	ACTAACATGTTTCAGCAAGCCACTGCAGGAGTCTCAC
MP_rev16	ACTACCCGGGTTAGCTTGAAGATGTACCAGTGCCTCC
malE_fw2	CGAGCAATTGACCAACAAGGACCATAGATTATGAAAATCCATCACCATCAC CATCACGAAGAAGGTAAACTGGTAATCTGG
malE_rv1	CAGCAGATCTTTGTTATAAATCAG
ARF5-DBD_fw1	CACCCCATGGGGGACAGTGACGAAATCTATGCTC
ARF5-DBD_rv1	GGTGCTCGAGTTAATCCGCTGAGAGAACTGATG

Mutagenesis	
NOS-mut_for1	GCCGAGTCAACCGTGTACGTCTCCCCCCGCC
NOS-mut_rev1	CGTACACGGTTGACTCGGCCGTCCAGTCGTAGGCG

qRT-PCR	
AtHB8for6	TCCAAATCAGAGGGGTTGAG
AtHB8rev6	CGACGCGATCACACTTCTTA
WOX4 qPCR F	CCTCCGGCGTCACTTCAG
WOX4 qPCR R	GGGTTCCACCTTGTCCCTC
PIN1for7	TGTTACTGTTCGTCGTTCTAATGC
PIN1rev7	ACCACCAGAAGCCATCATCG
EIF4A1for	ATCCAAGTTGGTGTGTTCTCC
EIF4A1rev	GAGTGTCTCGAGCTTCCACTC
qACT2f	GCCATCCAAGCTGTTCTCTC
qACT2r	ACCCTCGTAGATTGGCACAG

EMSA probes	
PIN1_fw	TCAAACTAAAGACAAAAGCTCT
PIN1_rv	AGAGCTTTTGTCTTTAGTTTGA
STG_fw	GCCGACAAAAAGTAACTAATAATTTGTAATTATTAGATGTAAGACAGGAG ATGAGATAGGGAGACAAGAG
STG_rv	CTCTTGTCTCCCTATCTCATCTCCTGTCTTACATCTAATAATTACAAATTAT TAGTTACTTTTTGTCGGC
WOX4a_fw	GCTCCATGCAGACATGAACATA
WOX4a_rv	TATGTTCATGTCTGCATGGAGC
WOX4a_mut_fw	GCTCCATGCAGCCATGAACATA
WOX4a_mut_rv	TATGTTCATGGCTGCATGGAGC
WOX4b_fw	AATCTGTCATTACCGACATAGT
WOX4b_rv	ACTATGTCGGTAATGACAGATT
CLV1_fw	CATCGTCGTCACGTGATGGG
CLV1_rv	CCCATCACGTGACGACGATG
