# Supplementary material for: Spatial specificity of auxin responses coordinates wood formation
Source: Nat Commun. 2018 Feb 28;9:875. doi: 10.1038/s41467-018-03256-2 (PMC5830446; doi:10.1038/s41467-018-03256-2)
Supplement: Supplementary file 3 — Description of Additional Supplementary Files [file 41467_2018_3256_MOESM3_ESM.pdf]

Description of Supplementary Files

File Name: Supplementary Data 1
Description: Genes responding to auxin signaling in the cambium.

File Name: Supplementary Data 2
Description: GO terms and categories of identified genes.
